# Structural basis for non-genuine phenolic acceptor substrate specificity of *Streptomyces roseochromogenes* prenyltransferase CloQ from the ABBA/PT-barrel superfamily

**DOI:** 10.1371/journal.pone.0174665

**Published:** 2017-03-29

**Authors:** Carla Araya-Cloutier, Bianca Martens, Gijs Schaftenaar, Franziska Leipoldt, Harry Gruppen, Jean-Paul Vincken

**Affiliations:** 1 Laboratory of Food Chemistry, Wageningen University, Wageningen, The Netherlands; 2 Nijmegen Centre for Molecular Sciences, Radboud University Medical Centre, Nijmegen, The Netherlands; 3 Pharmaceutical Biology, Pharmaceutical Institute, Eberhard Karls University Tübingen, Tübingen, Germany; University of Canterbury, NEW ZEALAND

## Abstract

Acceptor substrate specificity of *Streptomyces roseochromogenes* prenyltransferase SrCloQ was investigated using different non-genuine phenolic compounds. RP-UHPLC-UV-MSn was used for the tentative annotation and quantification of the prenylated products. Flavonoids, isoflavonoids and stilbenoids with different types of substitution were prenylated by SrCloQ, although with less efficiency than the genuine substrate 4-hydroxyphenylpyruvate. The isoflavan equol, followed by the flavone 7,4’-dihydroxyflavone, were the best non-genuine acceptor substrates. B-ring *C*-prenylation was in general preferred over A-ring *C*-prenylation (ratio 5:1). Docking studies of non-genuine acceptor substrates with the B-ring oriented towards the donor substrate dimethylallyl pyrophosphate, showed that the carbonyl group of the C-ring was able to make stabilizing interactions with the residue Arg160, which might determine the preference observed for B-ring prenylation. No reaction products were formed when the acceptor substrate had no phenolic hydroxyl groups. This preference can be explained by the essential hydrogen bond needed between a phenolic hydroxyl group and the residue Glu281. Acceptor substrates with an additional hydroxyl group at the *C*3’ position (B-ring), were mainly *O*3’-prenylated (> 80% of the reaction products). This can be explained by the proximity of the C3’ hydroxyl group to the donor substrate at the catalytic site. Flavones were preferred over isoflavones by SrCloQ. Docking studies suggested that the orientation of the B-ring and of the phenolic hydroxyl group at position *C*7 (A-ring) of flavones towards the residue Tyr233 plays an important role in this observed preference. Finally, the insights obtained on acceptor substrate specificity and regioselectivity for SrCloQ were extended to other prenyltransferases from the CloQ/NhpB family.

## Introduction

Prenylation is one of nature’s tools to modulate the bioactivity of primary [1] and secondary metabolites [2, 3] by increasing their lipophilicity and, thereby, their interactions with biological targets, such as proteins and membranes [4]. The enzymes responsible for transferring a prenyl group from a donor substrate (e.g. dimethylallyl pyrophosphate) to an acceptor substrate are known as prenyltransferases (PTs) [5]. Aromatic PTs catalyse the transfer reaction of prenyl moieties onto aromatic acceptors, such as phenolic acids, (iso)flavonoids, coumarins, naphthalenes, phenazines, and indole derivatives. These enzymes contribute substantially to the large diversity of secondary metabolites present in plants, fungi, and bacteria [6, 7].

Plants from the Fabaceae family are well known for their production of prenylated isoflavonoids upon abiotic or biotic stress [8]. The prenyl group is most often added to a free aromatic carbon (*C*-prenylation), but also to phenolic oxygens (*O*-prenylation) [9]. Several reports on legume PTs are available. PTs responsible for the production of glyceollins in soybean (*Glycine max* L.) and phaseollin in kidney bean (*Phaseolus vulgaris* L.) are localized in the membrane of plastids. Solubilisation of these PTs required detergents, which negatively affected enzyme activity and stability [10]. Furthermore, characterization of *Sophora flavescens* PT revealed donor and acceptor specificity to be confined to the known genuine substrates (i.e. *in vivo* substrates) [11]. An overview of recently characterized plant PTs can be found elsewhere [12].

Contrary to plant PTs, microbial PTs appear to be attractive biotechnological tools as most of them are soluble, i.e. not membrane-bound [4] and can potentially be obtained in significant amounts for the *in vitro* production of novel and bioactive prenylated compounds [13]. In the last decade, a new superfamily of soluble aromatic PTs isolated from microorganisms was discovered. This superfamily, named ABBA, has been considered for enhancement of molecular diversity and bioactivity of natural compounds due to their promiscuity for different non-genuine acceptor substrates [14]. The ABBA superfamily is a group of enzymes with a unique type of PT barrel fold comprising a series of 5 repetitive ααββ elements [13, 15]. More specifically, this PT-barrel is formed of 10 antiparallel β strands forming a spacious central solvent-filled cavity, where acceptor and donor substrates bind, surrounded by a ring of 10 α-helices [16]. Structural analysis of these PTs has revealed that there is a tendency for polar residues to cluster into the top half of the cavity where the pyrophosphate isoprenoid donor substrate binds. Non-polar residues cluster in the lower half of the cavity where the acceptor substrate binds [17]. Assays with microbial PTs have revealed broad aromatic acceptor substrate specificity, while often preserving donor substrate selectivity [18, 19].

Phylogenetic analysis of the ABBA PT superfamily revealed two distinct homologous families: one comprises the indole PTs, i.e. the DMATS/CymD family; the other one comprises the phenol/phenazines PTs, i.e. the CloQ/NphB family [14, 20]. Within the CloQ/NphB family there are PTs involved in meroterpenoid and prenylated phenazine biosynthesis (e.g. NphB, Fnq26, SCO7190, PpzP and Fur7) and PTs involved in novobiocin and clorobiocin biosynthesis (i.e. NovQ and CloQ) [4, 13].

**Fig 1:**
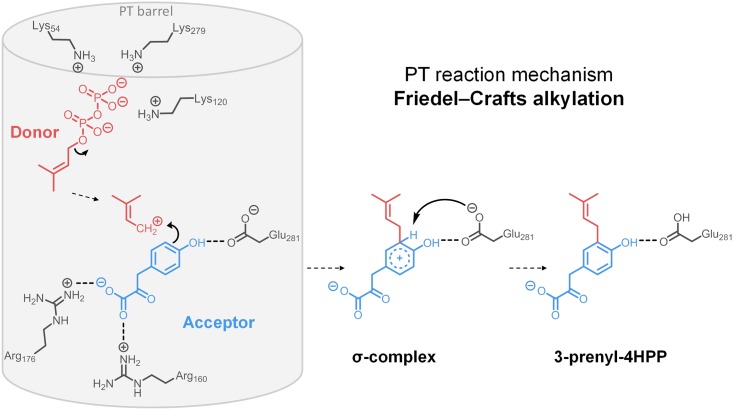
Schematic representation of the proposed mechanism of prenylation by *Sr*CloQ, adapted from previous work [17]. Important residues interacting with the donor and acceptor substrate are shown in grey colour. The donor substrate DMAPP is shown in red, whereas the genuine substrate is shown in blue.

*Streptomyces roseochromogenes* CloQ (*Sr*CloQ) was one of the first members of the ABBA superfamily discovered [15, 21]. *Sr*CloQ is an aromatic PT catalysing the *C*-prenylation (C_5_ isoprenoid unit) of 4-hydroxyphenylpyruvate (4-HPP), as part of the biosynthesis of the antibiotic clorobiocin [21]. More recent crystallization and simulation studies report key residues involved in substrate binding and on the mechanism of action of this enzyme (Fig 1). *Sr*CloQ is thought to perform a Friedel-Crafts type of alkylation of the acceptor 4-HPP via the formation of a carbocation on the prenyl donor. The electropositive nature of the upper cavity of the PT barrel helps to lower the energy barrier to catalysis by facilitating the cleavage of the C–O bond of the isoprenoid donor substrate resulting in the formation of a prenyl cation [17]. The prenyl cation, stabilized by charge delocalization, performs an electrophilic attack on the *C*3 of the aromatic ring of the acceptor substrate, resulting in an intermediary positive σ complex. The residue Glu281 is ideally placed in the active site to make a hydrogen bond with the phenolic hydroxyl group of 4-HPP and to neutralize the σ complex by proton abstraction. The residues Arg160 and Arg176 form salt bridges with the carboxyl group of 4-HPP, which is thought to be important for positioning the substrate [22]. The residues Glu281 and Arg160 proved to be essential for good catalytic activity, while other residues (e.g. Cys215, Cys297) stabilise binding of the aromatic substrates in the active site.[17, 22]

To date, there have been no studies on the acceptor substrate specificity of *Sr*CloQ regarding phenolic compounds, such as flavonoids, isoflavonoids and stilbenoids. Other studies with closely related PTs (e.g. *Streptomyces spheroids* NovQ) have reported the substrate specificity with phenolic compounds as acceptor substrates [18, 19, 23], but have not studied in detail the structural basis for non-genuine substrate specificity. In order to provide insight on the structural basis for prenylation of phenolic compounds, (iso)flavonoid and stilbenoid substrate specificities of *Sr*CloQ were determined by *in vitro* enzymatic assays and analysis of reaction products by means of RP-UHPLC-UV-MS^n^. Furthermore, we analysed *in silico* the interactions between the phenolic acceptors and *Sr*CloQ’s active site, and propose the interactions between the phenolic acceptor substrates and the active site residues, governing the substrate specificity experimentally observed. In addition, we compared the phenolic substrate specificity and regioselectivity of *Sr*CloQ with that of other members of the CloQ/NphB family. We hypothesized that (iso)flavonoids will be prenylated by *Sr*CloQ at the B-ring due to the similarities of this ring with the phenolic ring of *Sr*CloQ’s genuine substrate 4-HPP.

## Materials and methods

### Chemicals

The construction of the plasmid containing *Sr*CloQ has been described elsewhere [21]. Daidzein, genistein, 4-hydroxyphenylpyruvate (4-HPP) dimethylallyl pyrophosphate (DMAPP, donor substrate), growth media (lysogeny broth (LB) and terrific broth (TB)) and antibiotics (kanamycin and chloramphenicol) were obtained from Sigma Aldrich (St. Louis, MO, USA). 3'-Hydroxy-daidzein was obtained from Alfa Aesar (Ward Hill, MA, USA) and all other pure (iso)flavonoids and resveratrol were obtained from ICC Chemical Corporation (New York, NY, USA). Acetonitrile (ACN; ULC/MS grade), water acidified with acetic acid (HOAc, 0.1% v/v) (ULC/MS grade), and methanol (MeOH) (ULC/MS grade) were purchased from Biosolve (Valkenswaard, The Netherlands). Water for purposes other than UHPLC was prepared using a Milli-Q water purification system (Millipore, Molsheim, France). Glycerol was purchased from VWR International BV (Radnor, PA, USA). Other chemicals were purchased from Merck (Darmstadt, Germany) or Sigma Aldrich.

### Expression and purification of *Sr*CloQ

Production of the His-tagged *Sr*CloQ was based on previous studies [16, 21]. Calcium chloride transformation was performed with cells of *Escherichia coli* Rosetta (DE3) plysS (Promega, Madison, WI, USA). Cells harbouring *Srcloq* were cultured in TB medium supplemented with chloramphenicol (34 μg mL^-1^) and kanamycin (50 μg mL^-1^) for selection. Induction with isopropyl thiogalactoside (IPTG, Promega) was performed for 19 h at 20°C, after an OD_600nm_ of 0.6 was reached. After harvesting the cells, cells were resuspended in lysis buffer (2.5 mL lysis buffer per g cells; 50 mM Tris·HCl pH 8.0, 500 mM NaCl, 10% (v/v) glycerol, 10 mM β-mercaptoethanol, 1% (v/v) Tween 20, 20 mM imidazole, 0.5 mM phenylmethylsulfonyl fluoride, 0.5 mg mL^-1^ lysozyme). Lysis was performed by sonication (Sonifier S-250D, Branson, Danbury, CT, USA) in 9 sets of 30 s at an amplitude of 30% (pulse was alternatively turned on and off for periods of 10 s). The His-tagged *Sr*CloQ was purified from the cell lysate by affinity chromatography with an ÄKTA explorer system (GE Healthcare, Little Chalfont, UK), with a HisTrap HP 5 mL column (GE Healthcare) and buffers A (50 mM Tris-HCl pH 7.5, 20 mM imidazole) and B (50 mM Tris-HCl pH 7.5, 250 mM imidazole), at 5ml min^-1^. After equilibration of the column with buffer A, cell lysate was loaded and *Sr*CloQ was eluted with buffer B. The fractions containing the His-tagged protein were pooled, concentrated and desalted using Amicon Ultra-15 10K centrifugal filter devices (Merck Millipore, Billerica, MA, USA).

### Protein content and composition

Protein content was determined according to Bradford. A calibration curve was made with bovine serum albumin (BSA) in concentrations of 0.25–1.0 mg mL^-1^. Enzyme purity was confirmed by SDS-PAGE under reducing conditions on a Mini-protean II system (Bio-Rad Laboratories, Hercules, CA, USA), according to the manufacturer’s instructions. Commercially prepared mini-protean TGX gels (Bio-Rad) were used with Coomassie InstantBlue (Expedion, Cambridge, UK), with the marker Precision Plus Protein^™^ dual colour standards (Bio-Rad). Samples (8 μL) were loaded onto a gel, and the separation was done by applying 200 V for 45 min.

### Assay for PT activity

The reaction mixture contained: aromatic substrate (0.5 mM, 4-HPP, flavonoids, isoflavonoids or resveratrol), DMAPP (0.5 mM), NaCl (500 mM), glycerol (10% v/v), Tris·HCl buffer (pH 7.5, 100 mM), Mg^2+^ (7 mM in the form of MgCl_2_, for enhancement of activity) and purified *Sr*CloQ (30 μM). In analogy with previous studies on the *in vitro* prenylation of flavonoids with *Streptomyces* sp. strain CL190 (*Scl*NphB, 22% identity with *Sr*CloQ) [15, 18], incubation time of *Sr*CloQ with the non-genuine substrates was prolonged in comparison with that of the genuine substrate 4-HPP (24–48 h), due to the anticipated lower efficiency in conversion of the (iso)flavonoids. The mixtures were incubated for a maximum of 48 h at 30°C. The reaction was ended by adding ethyl acetate (400 μL) containing formic acid (0.5% v/v). The solution was vortexed and centrifuged (room temperature, 5 min, 10,000 *g*), after which the organic layer was evaporated and the residue was re-suspended in methanol (100 μL) for analysis on RP-UHPLC-UV-MS. Experiments were performed in duplicate.

### Analysis of reaction products

Reaction products were analysed by Ultra High Performance Liquid Chromatography and Mass Spectrometry (UHPLC-UV-MS). An Accela Velos UHPLC system (Thermo Scientific, San Jose, CA, USA) was equipped with a pump, autosampler and photodiode array (PDA) detector. Samples (1 μL) were loaded onto an Acquity UPLC BEH Shield RP18 column (2.1 i.d. mm x 150 mm, 1.7 μm particle size; Waters, Milford, MA, USA) with an Acquity UPLC BEH Shield RP18 VanGuard pre-column (2.1 i.d. mm x 5 mm, 1.7 μm particle size; Waters).

Water containing HOAc (0.1% v/v) and ACN (1% v/v), eluent A, and ACN containing HOAc (0.1% v/v), eluent B, were used as solvents at a flow rate of 300 μL min^-1^. The following elution profile was used: 1 min isocratic at 9% v/v B; 1.5 min linear gradient from 9–25% B; 7 min linear gradient from 25–50% B; 3 min isocratic on 50% B; 10 min linear gradient from 50–100% B; 2 min isocratic on 100% B, 1 min linear gradient from 100–9% B. Column temperature was set at 40°C and PDA detector was set to measure from 200–600 nm.

Mass spectrometric (MS) analysis was performed on a LTQ Velos (Thermo Scientific), which was equipped with a heated ESI-MS probe coupled to the RP-UHPLC. Full scan MS was performed in both negative ionisation (NI) and positive ionisation (PI) mode, in which data were acquired in a *m/z* range of 90–1500 Da. For tentative annotation, data-dependent MS^n^ analysis on the most intense (product) ion was performed with normalised collision energy of 35%. Prenylated products were monitored by single ion monitoring (SIM) scanning mode followed by single reaction monitoring (SRM) on the most abundant fragment ions. The system was tuned with genistein in PI and NI mode via automatic tuning using Tune Plus (Xcalibur v.2.2, Thermo Scientific). Nitrogen was used as sheath and auxiliary gas. The ITT temperature was 400°C and the source voltage was 3.50 kV (NI) or 4.50 kV (PI).

The tentative annotation of prenylated reaction products was performed by means of Xcalibur (version 2.2., Thermo Scientific). The position of the prenyl group within the phenolic skeleton (i.e. A- or B-ring) was elucidated by analysis of the *retro*-Diels-Alders (RDA) fragments in PI [24]: when the C-ring of (iso)flavonoids was cleaved in MS^3^, one of the remaining fragments still contained one carbon reminiscent of the prenyl chain (split in MS^2^), which can be used to diagnose the ring at which the prenyl was attached [25].

Quantification of phenolic compounds was performed using the following equation Eq (1) [26], derived from the Lambert-Beer’s law:

$$C=\frac{area\;\times \;Q}{\varepsilon \;\times \;l\;\times \;V_{inj}\;\times k_{cell}}$$ (1)

in which *C* is concentration (M), *area* is the integrated area of the UV peak at the specific wavelength (AU·s), *Q* is the flow rate (5 μL s^-1^), *ε* is the molar extinction coefficient (AU M^-1^·cm^-1^), *l* is the path length of the UV cell provided by the manufacturer (5 cm), *V*_*inj*_ is the injected volume of sample (1 μL), and *k*_*cell*_ is a constant related to the cell geometry of the UV detector [26]. This equation relates the duration of absorbance given by the UHPLC system (AU·s) to an actual absorbance value (AU) for the Lambert-Beer’s equation. The *k*_*cell*_ represents the correction factor for the absorption of light by the coating material of the flow cell. The *k*_*cell*_ (0.82 ± 0.09) was determined with standard solutions of daidzein (248 nm), genistein (263 nm) and resveratrol (310 nm) (with five concentrations each, in the range of 0.001–0.1 mg mL^-1^). The *ε* of the prenylated reaction products was assumed to be the same as that of the non-prenylated substrate, as shown in Table A in S1 File. The percentage of conversion of the different aromatic substrates was calculated as the μmoles of prenylated products formed from the initial aromatic substrate concentration (500 μM), multiplied by 100.

### *In silico* modelling

Molecular Operating Environment (MOE), 2013.08 (Chemical Computing Group, Montreal, QC, Canada) was used to analyse the PT structure and to perform docking studies. The *Sr*CloQ model (Protein Database entry 2XLQ) with the genuine substrate (4-HPP) bound to the active site was used for the docking studies [17]. The location of the DMAPP substrate inside the *Sr*CloQ active site was modelled based on the position of the geranyl pyrophosphate (GPP) inside *Scl*NphB (PDB 1ZB6) [27]. The placement of DMAPP was achieved by first creating a 3D alignment of the 2XLQ and 1ZB6 structures and subsequently taking the GPP position and transferring it to the 2XLQ structure, followed by molecular editing to convert GPP into DMAPP. Finally, a local geometry optimization was performed, at which only DMAPP was kept flexible. Further refinement of the DMAPP and 4-HPP position was performed using MMFF94x energy minimization in MOE. The *LigX* module in MOE was used as a guide to confirm that the placement was in line with that of the GPP inside 1ZB6. In accordance with previous literature [17, 22], our modelled negatively charged donor substrate DMAPP (Figure A in S1 File) made interactions with the positively charged residues Lys54, Arg66, Lys 279, and Lys120, and with the aromatic residues Tyr233 and Tyr174. The genuine substrate 4-HPP made the essential hydrogen bond with the residue Glu281, as well as with Arg160 and Arg176.

The 3D structures of the phenolic compounds were built with MOE and MMFF94x energy minimization (gradient 0.01) was performed for all molecules. Induced fit was used as docking mode and the predicted pose was selected based on the dock score (*S*) as implemented in MOE.

### Sequence and structure comparison

Alignment and sequence identity analysis of PTs from the CloQ/NphB PT family was performed with UniProt (http://www.uniprot.org/align/) and visualized with ESPript 3.0 [28]. Structure superposition of *Sr*CloQ and *Scl*NphB was performed with MOE.

## Results

### Enzyme activity of *Sr*CloQ

*Sr*CloQ was expressed as His-tagged protein in *E*. *coli* and purified by Ni^2+^ affinity chromatography (Figure B in S1 File). The purified enzyme showed the expected molecular mass (35 kDa) [21] and an estimated purity of >90% according to the SDS-PAGE analysis (Figure C in S1 File). Activity of *Sr*CloQ was confirmed in control experiments with its genuine substrate (4-HPP); incubations without *Sr*CloQ did not yield prenylated products (Figure D in S1 File).

**Fig 2:**
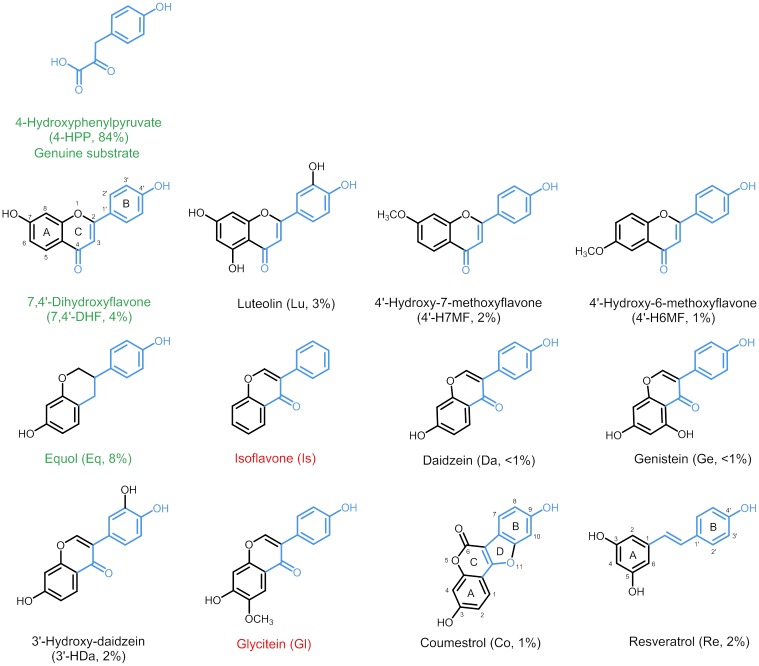
Aromatic acceptor substrates and their percentage of conversion (molar %) by *Sr*CloQ. The atoms shared with the genuine substrate are highlighted in blue colour. Phenolic substrates with green label represent the best acceptor substrates, whereas those with red labels were not utilized by the enzyme.

Fig 2 shows the structure of all aromatic acceptor substrates tested, i.e. flavonoids, isoflavonoids and the stilbenoid resveratrol, as well as the percentage of molar conversion by *Sr*CloQ. Compounds had different types of substituents, such as hydroxyl, methoxyl and carbonyl groups, at different positions of the skeleton. For optimal visualization, the structures of compounds are oriented with the B-ring of the phenolics in the same direction as the phenol ring of the genuine substrate 4-HPP. *Sr*CloQ showed promiscuity in that it was able to use most phenolics provided as acceptor substrates, i.e. we found reaction products for 10 out of 12 phenolic compounds tested. As expected, conversion of the genuine substrate 4-HPP was more efficient (>80% conversion) in comparison to those of the other phenolic test substrates (< 10% conversion) under the conditions used. Of all phenolics tested, the isoflavan equol and the flavone 4’,7-dihydroxyflavone were the best substrates for prenylation. Daidzein and genistein were converted only in very small amounts, whereas isoflavone (no hydroxyl groups present) and glycitein (methoxyl group at position *C*6) were not converted at all.

### Structure elucidation of reaction products

Table 1 shows the list of reaction products tentatively annotated by means of UHPLC-UV-MS^n^. Prenylation of substrates was confirmed by the neutral loss of 56 Da [C_4_H_8_] or 68 Da [C_5_H_9_] in NI and/or PI mode [29]. For more in-depth structural elucidation, tandem MS, single-ion-monitoring (SIM) and single-reaction-monitoring (SRM) scan modes were used to tentatively annotate the reaction products with respect to A- or B-ring prenylation and *C*- or *O*-prenylation.

**Table 1:** *Sr*CloQ reaction products tentatively annotated by RP-UHPLC-UV-MS^n^.

Substrate	Conversion (% ± std)	No.	RT (min)	λ_max_ (nm)	[M-H]^-^	MS^2^ (rel. abundance)	MS^3^ (rel. abundance)	[M+H]^+^	MS^2^ (rel. abundance)	MS^3^ (rel. abundance)	Tentative annotation
4-HPP	84 ± 15	**1**	7.8	280	189	134 (100), 174 (10)	106 (100)	n.d. ^a^			*C*-prenyl-4-HBAL ^b^
		**2**	8.9	n.d.	189	134 (100), 174 (10)	105 (100), 106 (30)	n.d.			*C*-prenyl-4-HBAL
		**3**	9.1	n.d.	189	134 (100), 174 (10)	106 (100)	n.d.			*C*-prenyl-4-HBAL
		**4**	9.9	278	219	175 (100), 201 (10)	132 (100),107 (90), 119 (70), 106 (40), 157 (30)	n.d.			*C*-prenyl-4-HPA ^b^
		**5**	10.1	278	219	175 (100)	107 (100), 132 (80), 119 (60), 106 (50), 157 (40)	n.d.			*C*-prenyl-4-HPA
		**6**	11.4	280	189	134 (100), 174 (10)	106 (100)	n.d.			*C*-prenyl-4-HBAL
		**7**	12.6	285	189	134 (100), 174 (10)	106 (100)	n.d.			*C*-prenyl-4-HBAL
Eq	7.7 ± 1.4	**8**	16.9	n.d.	309	121 (100), 187 (70), 135 (30)	92 (100), 77 (25), 65 (15)	311	123 (100), 175 (90), 255 (50), 189 (40), 149 (15), 201 (10)	95 (100), 67 (35), 77 (10)	B_ring_-*C*-prenyl-Eq
		**9**	17.1	285	309	189 (100), 203 (20), 119 (10)	134 (100)	311	191 (100), 205 (50), 255 (20), 107 (10)	173 (100), 149 (50), 145 (10), 137 (10), 135 (10)	A_ring_-*C*-prenyl-Eq ^e^
7,4'-DHF	4.2 ± 1.2	**10**	14.5	334	321	266 (100), 265 (10)	237 (100), 223 (90), 222 (70), 238 (70), 265 (30), 135 (20)	323	267 (100), 268 (15)	239 (100), 213 (80), 240(20), 228(20)	B_ring_-*C*-prenyl-7,4’-DHF
Lu	3.2 ± 0.6	**11**	13.4	n.d.	353	151 (100), 284 (95), 283 (90), 201 (70), 335 (30), 324 (20)	n.d.	355	n.d.	n.d.	*C*-prenyl-Lu
		**12**	14.4	n.d.	353	151(100), 298 (50), 201 (30), 231 (20), 335 (15)	107 (100), 83 (10)	355	n.d.	n.d.	B_ring_-*C*-prenyl-Lu
		**13**	16.5	266, 348	353	284 (100)	256 (100)	355	287(100)	153 (100), 287 (90), 259 (50), 245 (30), 241 (30), 161 (20)	*O*-prenyl-Lu
4'-H-7-MF	1.7 ± 0.3	**14**	16.6	334	357, *335* ^c^	342 (100), 343 (20), 207 (10)	342 (100), 341 (90), 343 (40), 325 (30), 314 (20), 205 (20)	359, *337* ^c^	281 (100), 344 (30), 253 (30), 282 (20)	253 (100), 227 (25), 254 (20)	B_ring_-*C*-prenyl-4'-H-7-MF
		**15**	17.2	335	335	280 (100), 320 (80), 277 (60), 265 (15)	n.d.	337	281 (100), 282 (15)	253 (100), 227 (90), 254 (20), 228 (15)	B_ring_-*C*-prenyl-4'-H-7-MF
		**16**	18.2	327	357, *335* ^c^	n.d.	n.d.	359, *337* ^c^	268 (100), 331 (30), 269 (20)	240 (100), 241 (20)	4’-*O*-prenyl-7-MF
3-HDa	1.7 ± 0.4	**17**	10.9	n.d	337	268 (100), 201 (25), 309 (10), 293 (10), 135 (10)	n.d.	339	n.d.		B_ring_-*C*-prenyl-3'HDa
		**18**	12.8	n.d	337	268 (100), 281 (40), 309 (10)	n.d.	339	n.d.		prenyl-3'HDa
		**19**	13.2	260	337	268 (100)	n.d.	339	271 (100), 283 (10)	137 (100), 243 (90), 253 (85), 215 (80), 225 (80), 161 (20), 181 (15), 201 (10)	*O*-prenyl-3'HDa
Re	1.6 ± 0.3	**20**	13.5	n.d.	295	240 (100), 251 (80), 235 (50), 253 (35), 225 (30)	n.d.	n.d.			A_ring_-*C*-prenyl-Re
		**21**	14.2	323	295	240 (100), 251 (60), 253 (30), 225 (30)	195 (100), 212 (75), 170 (70), 225 (50), 197 (50)	n.d.			B_ring_-*C*-prenyl-Re
		**22**	16.6	320	295	226 (100)	n.d.	n.d.			*O*-prenyl-Re
Co	0.8 ± 0.3	**23**	18.5	349	335	279 (100), 280 (25), 292 (20)	251 (100), 279 (40), 223 (20) 252 (20), 280 (10)	337	269 (100), 270 (15), 281 (10)	241 (100), 225 (30), 197 (25), 242 (20)	*C*-prenyl-Co
		**24**	19.6	n.d.	335	266 (100), 279 (10)	n.d.	337	n.d.		*O*-prenyl-Co
4'-H-6-MF	0.8 ± 0.2	**25**	17.2	330	357, *335* ^c^	342 (100), 343 (20)	298 (100), 314 (30), 251 (25)	359, *337* ^c^	281 (100), 344 (40), 282 (20), 316 (20), 253 (15)	253 (100), 254 (20), 225 (10), 242 (10)	B_ring_-*C*-prenyl-4'-H-6-MF
		**26**	17.4	n.d.	357, *335* ^c^	n.d.		359, *337* ^c^	n.d.		prenyl-4’-H-6-MF
		**27**	18.6	n.d.	357, *335* ^c^	342 (100), 343 (20)		359, *337* ^c^	n.d.		prenyl-4’-H-6-MF
Da	0.6 ± 0.2	**28**	15.0	n.d.	321	265 (100), 266 (60),278 (15), 252 (10)	n.d.	323	267 (100), 268 (10), 255 (10)	239 (100), 240 (15), 211 (15), 137 (10)	B_ring_-*C*-prenyl-Da
Ge	0.4 ± 0.1	**29**	17.7	263	337	281 (100), 282 (30), 293 (15)	n.d.	n.d.			B_ring_-*C*-prenyl-Ge
		**30**	17.9	266	337	282 (100)	n.d.	n.d.			A_ring_-*C*-prenyl-Ge
		**31**	19.0	n.d.	337	268 (100), 255 (10)	n.d.	n.d.			*O*-prenyl-Ge
Gl	n.p. ^d^										
Is	n.p.										

^*a*^ Not determined (n.d.).

^*b*^ Under alkaline conditions 4-HPP decomposes to 4-Hydroxybenzaldehyde (4-HBAL) or 4-hydroxyphenylacetic acid (4-HPA).[21]

^*c*^ Parent ion formed a sodium adduct. The italic *m/z* represents the [M-H]^-^ or [M+H]^+^ ion.

^*d*^ No products formed (n.p.).

^*e*^ Based on previous studies on the MS fragmentation of standard isoflavones[25] we proposed this product to be *C*8-prenyl-equol.

#### A- or B-ring prenylation

Analysis of the *retro*-Diels-Alder (RDA) fragment ions from prenylated isomers in both NI and PI mode was used to determine the position of the prenyl substituents (A- or B-ring). The formation of the RDA fragments upon cleavage of the C-rings of (iso)flavonoids leave (part of) the prenyl group attached to the phenolic ring, which results in diagnostic fragments [25]. The most common bonds in (iso)flavonoids split, resulting in A-ring and B-ring containing ions, are the 1/3, 2/3, 0/2, 0/3, 0/4 or 2/4 bonds of the C-ring [24].

**Fig 3:**
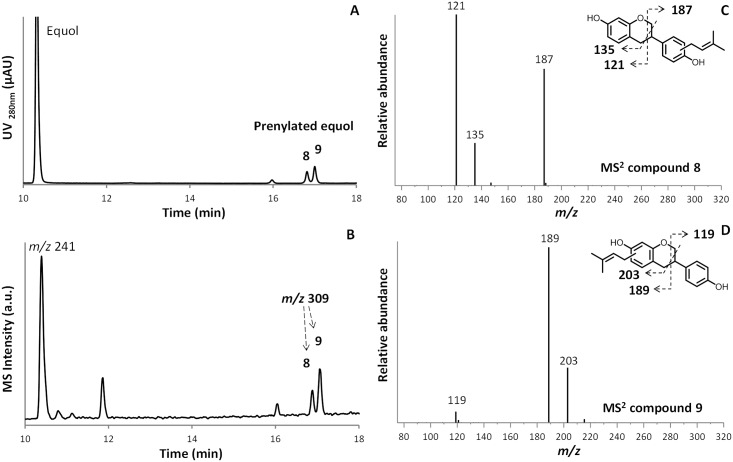
*Sr*CloQ prenylated equol products. RP-UHPLC-UV (**A**) and MS in NI (**B**) profiles of equol and prenylated equol products (products **8** and **9** in Table 1) after incubation of equol with DMAPP and *Sr*CloQ. MS^2^ spectra of *C*-prenylated equol (*m/z* 309) at retention times 16.9 (**8**, panel **C**) and 17.1 min (**9**, panel **D**). The proposed RDA fragmentation pathways are shown as inset.

Fig 3A and 3B show the UV and MS in NI mode chromatograms of the reaction products of equol incubated with *Sr*CloQ. The two main prenylated products formed eluted at 16.9 min (peak **8**) and 17.1 min (peak **9**). Fig 3C and 3D show the MS^2^ spectra of these prenylated equol isomers. Peak **8** showed high abundances of ions with *m/z* 121, 187 and 135. These values matched the RDA fragments of B-ring prenylated equol, i.e. ^1,3^A^-^, ^1,3^B^-^ and ^2,3^A^-^, respectively, as shown by the fragmentation pattern (Fig 3C). Peak **9** had different main *m/z* values (189, 203 and 119). These ions were formed by the same fragmentation pathways as described before, but now the RDA fragments corresponded with A-ring prenylated equol (Fig 3D).

#### *C*- or *O*-prenylation

**Fig 4:**
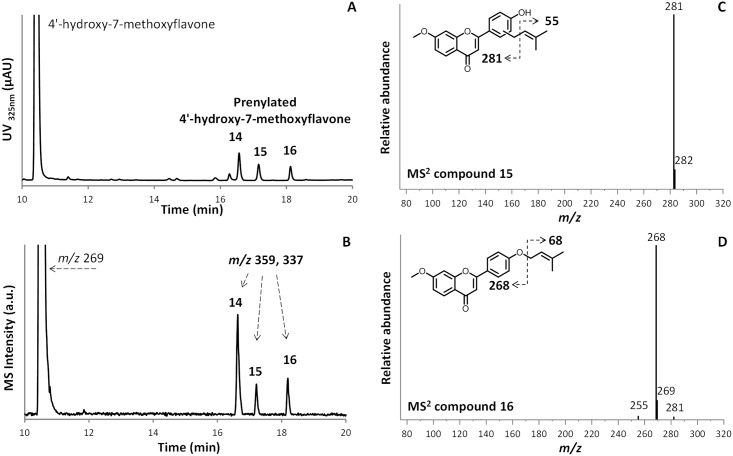
*Sr*CloQ prenylated 4’-hydroxy-7-methoxyflavone products. RP-UHPLC-UV (**A**) and MS in PI (**B**) profiles of 4’-hydroxy-7-methoxyflavone and prenylated products (**14**, **15** and **16** in Table 1) after incubation of 4’-hydroxy-7-methoxyflavone with DMAPP and *Sr*CloQ. MS^2^ spectra of *C*-prenylated isomer (*m/z* 359, Na adduct) at retention time 17.1 min (**15**, panel **C**) and *O*-prenylated isomer at 18.1 min (**16**, panel **D**). The proposed RDA fragmentation pathway is shown as inset.

Tandem MS analysis was used to distinguish *C*- and *O*-prenylation of the aromatic substrates. Fig 4A and 4B show the UV and MS in PI mode chromatograms of the prenylated products of 4’-hydroxy-7-methoxyflavone produced by *Sr*CloQ. Three main prenylated isomers were found. Two distinct fragmentation patterns could be distinguished for these isomers. Peak **14** and **15** showed [M+1–56]^+^ as main fragment (Fig 4C for peak **15**; see Table 1 for peak **14** fragmentation). Peak **16** showed [M+1–68]^+^ as the main fragment (Fig 4D). The prenyl group generates the fragment [M+H-56]^+^ when it is attached to a carbon of an aromatic ring [29], as in the MS^2^ of peak **14** and **15**. When it is attached to an oxygen of an aromatic ring, the prenyl group will split off intact ([M+H-68]^+^) [16], leaving the original aromatic substrate as main daughter ion, as in the MS^2^ spectrum of peak **16**. Moreover, the higher retention time of peak **16** in comparison with peak **14** and **15** on the reversed phase column, reflects that peak **16** is less polar than the other two, supporting our tentative annotation of *O*-prenylation of this reaction product.

In cases where *O*-prenylation could occur at both the A- and the B-ring (contrary to 4’-hydroxy-7-methoxyflavone, which has only one OH group available), the annotation of the A- or B-ring position of the prenyl group was not possible, because the prenyl detached completely in MS^2^, leaving no footprints to annotate the ring position.

### SrCloQ favours B-ring *C*-prenylation of (iso)flavonoids and stilbenoids

**Fig 5:**
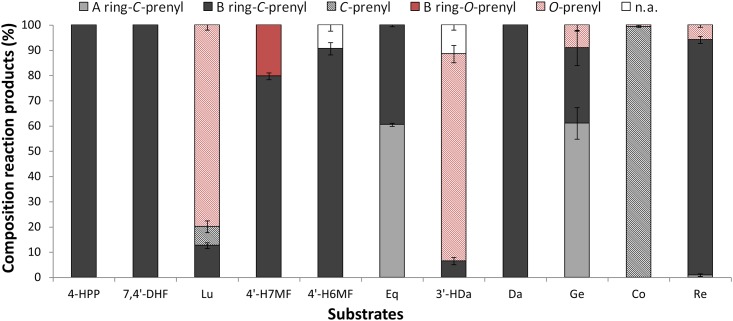
Molar composition of the prenylated products obtained with *Sr*CloQ. Error bars represent the standard deviation; *n*.*a*., not annotated.

For all phenolic substrates tested with *Sr*CloQ, the prenylated products obtained were annotated using the above rationale. Fig 5 shows the composition of the mixture of prenylated isomers formed by *Sr*CloQ. The enzyme favoured B-ring *C*-prenylation of substrates in aromatic rings. Considering the regioselectivity of *Ss*NovQ (84% sequence identity with *Sr*CloQ) towards daidzein and genistein [23], we postulate that the *C*3’ (B-ring) is the preferred position for prenylation by *Sr*CloQ. The only substrates that were A-ring prenylated were equol and genistein, albeit the latter in minute amounts. It was not possible to annotate the ring position of the prenyl group in coumestrol, as coumestrol does not undergo typical RDA fragmentation [30, 31]. Consequently no apparent A- or B-ring fragment ions were formed in MS^n^. Additionally, A-ring and B-ring prenylated coumestrol have been shown to yield the same fragmentation pattern in MS [32, 33]. Resveratrol does not show RDA fragmentation due to the lack of the C-ring. Thus, reaction products of resveratrol were tentatively annotated based on the fragmentation behaviour of (prenylated) resveratrol previously reported [34, 35].

*Sr*CloQ was able to *C*- or *O*-prenylate an acceptor molecule, such as luteolin, 4’-hydroxy-7-methoxyflavone, 3’-hydroxydaidzein, and genistein. Luteolin and 3’-hydroxydaidzein were primarily *O*-prenylated (≥ 80% of the reaction products). These two substrates were the only ones with an extra phenolic hydroxyl group attached to the B-ring (*meta*-hydroxyl group). The presence of hydroxyl groups proved to be essential for catalysis, as isoflavone (no hydroxyl groups) yielded no reaction products with *Sr*CloQ. Furthermore, the number of hydroxyl groups on (iso)flavonoids proved to have an effect on the enzyme activity. *Sr*CloQ yielded slightly higher quantity of reaction products with 7,4’-dihydroxyflavone (2 hydroxyl groups) compared to luteolin (4 hydroxyl groups). Methoxylated substrates resulted in lower conversion yields than their non-methoxylated derivatives, as for 4’-hydroxy-7-methoxyflavone compared to 4’,7-dihydroxyflavone.

## Discussion

### Mechanism behind non-genuine acceptor substrate specificity of *Sr*CloQ

To verify the interactions of the acceptor substrates tested with the protein, we docked all the molecules, including the genuine substrate 4-HPP, into the active site. Our docked 4-HPP made the essential interactions [22] with the residues Glu281 and Arg160, as well as with Arg176 and Cys297 (S1 Fig).

Using this model, we docked the different phenolic substrates tested into the active site cavity of *Sr*CloQ. In principle, the active site had room to accommodate all acceptor substrates tested, as no clashes were observed, not even for unreacted compounds such as glycitein. This observation is in accordance with simulation analysis done previously [22], which showed that the active site is large enough to accommodate flavonoids.

**Fig 6:**
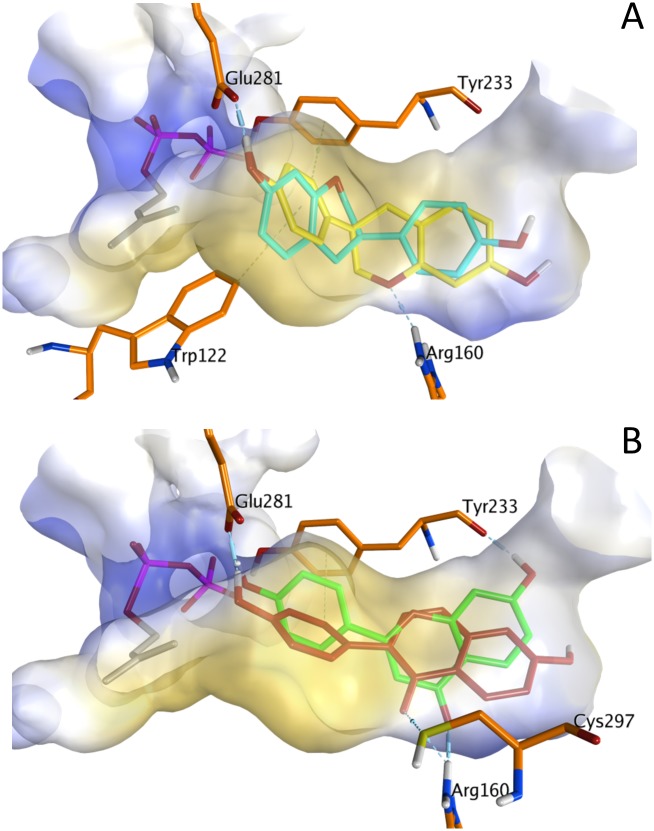
Phenolic aromatic substrates docked in the active site of *Sr*CloQ (PDB 2XLQ). (**A**) Comparison of equol with the A-ring (light blue) and B-ring (yellow) oriented towards the prenyl donor. (**B**) 7,4’-Dihydroxyflavone (green) and daidzein (dark red) with their B-ring towards the prenyl donor. Protein surface is coloured according to the lipophilic potential using MOE software default’s setting: yellow being lipophilic and blue hydrophilic (cut-off of 2.5). Donor substrate DMAPP is shown in gray with the phosphate group in pink. Residues that interact with the acceptor substrates are shown in orange: Glu281 anchors the substrate by H-bonding with the phenolic hydroxyl group; Arg160 stabilizes binding by H-bonding with the carbonyl or ether group of the C-ring; Tyr233 aromatic ring can make H-π bonds or π stacking interactions with the aromatic rings of the phenolic substrates, while its carbonyl group can make H-bonding with phenolic hydroxyl groups; Cys297 thiol group can interact with the carbonyl group of the phenolic substrate.

The best aromatic substrate tested was equol, which was prenylated at the A- or the B-ring. Upon docking of equol into the active site of *Sr*CloQ (Fig 6A), both the A- and B-ring can orient towards the donor substrate and make the hydrogen bond between the phenolic hydroxyl group and Glu281. Additionally, the aromatic ring can make H-π interactions with Tyr233 and Trp122, whereas the tetrahydropyran (C-ring) can interact with Arg160. These interactions are likely to help stabilizing the binding of equol inside the active site. Furthermore, this isoflavan is one of the smallest acceptor substrates tested, and it is more amenable to torsion than other isoflavonoids due to the lack of the *C*2–*C*3 double bond. Finally, it has less space limitations inside the active site due to the absence of the *C*4 carbonyl group. This might explain the promiscuity of *Sr*CloQ to prenylate both the A- and the B-ring of equol.

In addition, we studied the interactions of the second best acceptor substrate (i.e. 7,4’-dihydroxyflavone) with *Sr*CloQ and compared them with those of its isoflavone isomer and one of the worst acceptor substrates (i.e. daidzein) (Fig 6B). We found that 7,4’-dihydroxyflavone can form the crucial hydrogen bond between the phenolic hydroxyl group at the B-ring and the residue Glu281. Furthermore, the *C*4 carbonyl group interacts with the anchoring residue Arg160. Also, the aromatic residue Tyr233 is able to make a hydrogen bond with the *C*7 hydroxyl group in the A-ring and π stacking interactions with the B-ring of the flavone. Tyr233 has been reported to stabilize the DMAPP substrate by hydrogen bonding and to make van der Waals interactions with 4-HPP [17, 22].

Daidzein, which is the isoflavone isomer of 7,4’-dihydroxyflavone, also showed the essential interaction between the *C*4’ hydroxyl group and Glu281. Furthermore, the carbonyl group of daidzein makes the interactions with Arg160 and with Cys297. In contrast to the flavone, due to the different orientation of the A- and B-rings, daidzein does not make any interactions with Tyr233.

Overall, our results show that Arg160 plays an important role in stabilizing (iso)flavonoids in the active site of *Sr*CloQ via hydrogen bonds with the carbonyl or ether group in the C-ring. Moreover, our docking studies revealed that interactions with Tyr233 might contribute to the flavone over isoflavone preference observed in this study with *Sr*CloQ, and also in a previous study with *Ss*NovQ [23]. The *C*7 hydroxyl group at the A-ring of isoflavones is far away from this residue and the B-ring is oriented in a different direction. Consequently, daidzein is unable to make the relevant contacts with Tyr233, as opposed to the flavone. Mutation studies are required to confirm this role of Tyr233 in flavonoid over isoflavone preference by the PTs *Sr*CloQ and *Ss*NovQ.

### Comparison of non-genuine substrate preferences of different ABBA prenyltransferases

Using the information obtained from the docking studies with *Sr*CloQ and sequence comparison (Figure E in S1 File), we can extend our knowledge of the non-genuine aromatic substrate preferences of other closely related ABBA PTs [4, 15]. Table 2 shows an overview of the sequence identity and main acceptor substrate preferences of *Sr*CloQ compared with other members of the CloQ/NphB PT family reported in literature. This information can be useful for the selection of appropriate acceptor substrates for the *in vitro* production of novel bioactive prenylated compounds.

**Table 2:** Summary of acceptor substrate preferences and regioselectivity of *Sr*CloQ and other closely related ABBA prenyltransferases.

	*Sr*CloQ	*Ss*NovQ[23]	*Sco*7190[18, 19]	*Scl*NphB[18]
**Sequence identity with *Sr*CloQ (%)**	**-**	**85**	**25**	**21**
Genuine acceptor substrate	4-HPP ^a^	4-HPP	1,6-DHN ^b^	1,6-DHN
Genuine donor substrate	DMAPP ^c^	DMAPP	DMAPP	GPP ^d^
**Non-genuine acceptor substrate preferences**				
Flavonoids	+	+	+/-	+
Isoflavonoids	+/-	+/-	-	+/-
Substrate with no OH groups	-	n.t.	n.t.	n.t.
Substrate with OCH_3_ groups	-	n.t.	n.t.	-
**Regioselectivity**				
A-ring prenylation	+/-	-	+	+
B-ring prenylation	+	+	-	-
*C*-prenylation	++	++	+	+
*O*-prenylation	+	+	-	++
Double prenylation	-	+^e^	-	+

^*a*^ 4-hydroxyphenylpyruvate.

^*b*^ 1,6-dihydroxynaphtalene.

^*c*^ dimethylallylpyrophosphate.

^*d*^ geranylpyrophosphate.

^*e*^ Only with a phenolic acid (i.e. 3,4-*O*-diprenyl-caffeic acid), but not with any of the (iso)flavonoids or stilbenoids tested in their study.[23]

#### Acceptor substrate specificity

For all four bacterial PTases, flavonoids were preferred over isoflavonoids. According to our docking results, interactions of Tyr233 with flavones contributes to this preference observed in *Sr*CloQ. This Tyr233 is conserved in *Ss*NovQ. The other two enzymes contain alanine in that position instead (Figure E in S1 File). By superposing the active sites of *Sr*CloQ and *Scl*NphB (data not shown) it was apparent that *Scl*NphB contains a tyrosine residue (Tyr216, also conserved in *Sco*7190), with its side chain at an equivalent position as Tyr233 of *Sr*CloQ. The side chains of these tyrosine residues were both reported to make equivalent interactions with the donor (i.e. stabilization of the carbocation) and genuine acceptor substrates (π or van der Waal contacts with the aromatic ring of the acceptor substrates) [17, 22, 36]. Due to its position and orientation, it seems unlikely that Tyr216 can interact with the non-genuine substrates in a similar way as Tyr233. Therefore, residues other than Tyr216 seem responsible for the non-genuine acceptor substrate preference observed with *Scl*NphB.

The presence of the *para-*hydroxyl group in 4-HPP is essential, as the substrate is anchored with this hydroxyl by a hydrogen bond to Glu281. The potential non-genuine acceptor substrate isoflavone (Fig 2) does not contain hydroxyl groups. This explains the lack of reaction products of *Sr*CloQ with this molecule as acceptor substrate. Glu281facilitates the formation of the prenylated σ-complex and it neutralizes it by proton abstraction (Fig 1) [17]. Glu281 is conserved in *Ss*NovQ and *Sco*7190, but not in *Scl*NphB. Previous studies on the mechanism of *Scl*NphB revealed that the genuine acceptor substrate 1,6-dihydroxynaphthalene makes contacts with Ser51 and the non-genuine acceptor substrate flaviolin with Gln295 [19]. These residues are likely to help position the acceptor substrates in *Scl*NphB, as Glu281 in the other PTs. In *Scl*NphB, a water molecule, instead of any particular residue, is the most likely to facilitate the proton extraction step, according to simulation studies [36].

The addition of methoxyl groups to the A-ring, as in glycitein, decreased or cancelled the activity of *Sr*CloQ. The same result was observed for *Scl*NphB with the methoxylated substrate pterostilbene [18]. The increase in molecular size by the addition of this bulky substituent possibly hampers the entrance to the active site of the PTs. The effect of hydroxylation on the activity of *Ss*NovQ, *Sco*7190 and *Scl*NphB and the effect of methoxylation of phenolics on the activity of *Ss*NovQ and *Sco*7190 has to our knowledge not been tested, but it is tempting to propose a similar trend on activity as observed with *Sr*CloQ.

#### A- or B-ring regioselectivity

*Sr*CloQ and *Ss*NovQ favour B-ring prenylation, whereas *Sco*7190 and *Scl*NphB prefer A-ring prenylation. This preference for either the B- or A-ring prenylation might be explained by the structure of their reported genuine substrates: *Sr*CloQ and *Ss*NovQ use 4-HPP as the genuine substrate, which contains one phenyl ring thereby resembling the B-ring of (iso)flavonoids. In contrast, *Sco*7190 and *Scl*NphB use 1,6-dihydroxynaphtalene, which contains two connected rings, resembling the A- and C-ring of (iso)flavonoids. In addition, the residue Arg160, which makes interactions with the carbonyl group of 4-HPP and of the C-ring of (iso)flavonoids (Fig 6), is conserved among the B-ring prenylating enzymes (Figure E in S1 File). This interaction is likely to assist in orienting the B-ring of the acceptor substrate towards the donor substrate, facilitating B-ring prenylation.

#### *C*- or *O*-prenylation

*Sr*CloQ and *Ss*NovQ were able to either *C*- or *O*-prenylate the same (iso)flavonoid substrate, although *C*-prenylation was predominant. *Sco*7190 did not *O*-prenylate any of the (iso)flavonoids substrates tested, whereas *Scl*NphB predominantly showed preference for *O*-prenylation of (iso)flavonoids. There are no studies explaining what exactly determines *C*- versus *O*- prenylation preference of these bacterial PTs. With regard to *Sr*CloQ, *O*-prenylation was preferred when the aromatic ring contained two neighbouring hydroxyl groups, as with luteolin and 3’-hydroxydaidzein. Previous biochemical studies with *Sr*CloQ and *Sco*7190 showed only *C*-prenylation of aromatic substrates [17, 18, 21], however, none of the acceptors tested had the neighbouring hydroxyl group. Based on our docking studies, we observed that the *C*3’ hydroxyl group can be close enough to the donor substrate in the catalytic centre and prone to electrophilic attack by the allyl cation. In contrast to *Sr*CloQ, *Ss*NovQ was able to B-ring *O*-prenylate many (iso)flavonoid substrates without the *C*3’ hydroxyl group. Further (crystallization) studies with *Ss*NovQ may provide insight to understand this difference.

With regard to the number of prenyl groups attached to the phenolic substrates used in this study, *Sr*CloQ produced only mono prenylated products, similarly to *Ss*NovQ and *Sco*7190. In contrast, *Scl*NphB has been reported to produce double prenylated stilbenoids, specifically 2,4-digeranyl-resveratrol [18]. This difference might be explained by the facts that the bottom part of the PT barrel of *Sr*CloQ is less accessible and significantly narrower than that of *Scl*NphB [17].

## Conclusions

In this study we demonstrated that: (i) *Sr*CloQ can prenylate aromatic substrates belonging to the (iso)flavonoid and stilbenoid classes; (ii) *Sr*CloQ is able to either *C*- or *O*-prenylate the same acceptor substrate; (iii) *Sr*CloQ showed a preference for *C*-prenylation at the B-ring of (iso)flavonoids, as hypothesized in the introduction; (iv) the addition of a *meta* hydroxyl group at the B-ring changes the preference to *O*-prenylation. The genuine substrate 4-HPP showed the highest conversion yield, followed by equol and 7,4’-dihydroxyflavone. We propose, using *in silico* modelling, the mechanisms by which the acceptor substrate specificity and regioselectivity observed with *Sr*CloQ, but also of related PTs, can be explained. This information can help to choose the appropriate acceptor substrate for a specific PT when tailoring novel prenylated phenolic compounds.

## Supporting information

S1 File Supporting information. (PDF)

## References

[1] Palsuledesai CC, Distefano MD. Protein prenylation: enzymes, therapeutics, and biotechnology applications. ACS Chem Biol. 2015;10(1):51–62. 10.1021/cb500791f 25402849 PMC4301080

[2] Simons R, Gruppen H, Bovee TFH, Verbruggen MA, Vincken J-P. Prenylated isoflavonoids from plants as selective estrogen receptor modulators (phytoSERMs). Food Funct. 2012;3(8):810–27. 10.1039/c2fo10290k 22684228

[3] van de Schans MGM, Vincken J-P, de Waard P, Hamers ARM, Bovee TFH, Gruppen H. Glyceollins and dehydroglyceollins isolated from soybean act as SERMs and ER subtype-selective phytoestrogens. J Steroid Biochem Mol Biol. 2016;156:53–63. 10.1016/j.jsbmb.2015.11.020 26655113

[4] Winkelblech J, Fan A, Li S-M. Prenyltransferases as key enzymes in primary and secondary metabolism. Appl Microbiol Biotechnol. 2015;99(18):7379–97. 10.1007/s00253-015-6811-y 26216239

[5] Liang PH, Ko TP, Wang AHJ. Structure, mechanism and function of prenyltransferases. Eur J Biochem. 2002;269(14):3339–54. 12135472 10.1046/j.1432-1033.2002.03014.x

[6] Heide L. Prenyl transfer to aromatic substrates: genetics and enzymology. Curr Opin Chem Biol. 2009;13(2):171–9. 10.1016/j.cbpa.2009.02.020 19299193

[7] Yazaki K, Sasaki K, Tsurumaru Y. Prenylation of aromatic compounds, a key diversification of plant secondary metabolites. Phytochemistry. 2009;70(15–16):1739–45. 10.1016/j.phytochem.2009.08.023 19819506

[8] Veitch NC. Isoflavonoids of the Leguminosae. Nat Prod Rep. 2013;30(7):988–1027. 10.1039/c3np70024k 23736284

[9] Khalivulla SI, Reddy BAK, Gunasekar D, Blond A, Bodo B, Murthy MM, et al A new di-*O*-prenylated isoflavone from *Tephrosia tinctoria*. J Asian Nat Prod Res. 2008;10(10):953–5. 19003614 10.1080/10286020802217630

[10] Welle R, Grisebach H. Properties and solubilization of the prenyltransferase of isoflavonoid phytoalexin biosynthesis in soybean. Phytochemistry. 1991;30(2):479–84.

[11] Sasaki K, Tsurumaru Y, Yamamoto H, Yazaki K. Molecular characterization of a membrane-bound prenyltransferase specific for isoflavone from *Sophora flavescens*. J Biol Chem. 2011;286(27):24125–34. 10.1074/jbc.M111.244426 21576242 PMC3129193

[12] Yang X, Jiang Y, Yang J, He J, Sun J, Chen F, et al Prenylated flavonoids, promising nutraceuticals with impressive biological activities. Trends Food Sci Technol. 2015;44(1):93–104.

[13] Saleh O, Haagen Y, Seeger K, Heide L. Prenyl transfer to aromatic substrates in the biosynthesis of aminocoumarins, meroterpenoids and phenazines: The ABBA prenyltransferase family. Phytochemistry. 2009;70(15–16):1728–38. 10.1016/j.phytochem.2009.05.009 19559450

[14] Bonitz T, Alva V, Saleh O, Lupas AN, Heide L. Evolutionary relationships of microbial aromatic prenyltransferases. PLoS One. 2011;6(11):1–8. 10.1371/journal.pone.0027336 PMC3227686 22140437

[15] Tello M, Kuzuyama T, Heide L, Noel JP, Richard SB. The ABBA family of aromatic prenyltransferases: broadening natural product diversity. Cell Mol Life Sci. 2008;65(10):1459–63. 10.1007/s00018-008-7579-3 18322648 PMC2861910

[16] Haagen Y, Unsöld I, Westrich L, Gust B, Richard SB, Noel JP, et al A soluble, magnesium-independent prenyltransferase catalyzes reverse and regular *C*-prenylations and *O*-prenylations of aromatic substrates. FEBS Lett. 2007;581(16):2889–93. 10.1016/j.febslet.2007.05.031 17543953 PMC2860617

[17] Metzger U, Keller S, Stevenson CEM, Heide L, Lawson DM. Structure and mechanism of the magnesium-independent aromatic prenyltransferase CloQ from the clorobiocin biosynthetic pathway. J Mol Biol. 2010;404(4):611–26. 10.1016/j.jmb.2010.09.067 20946900

[18] Kumano T, Richard SB, Noel JP, Nishiyama M, Kuzuyama T. Chemoenzymatic syntheses of prenylated aromatic small molecules using *Streptomyces* prenyltransferases with relaxed substrate specificities. Bioorg Med Chem. 2008;16(17):8117–26. 10.1016/j.bmc.2008.07.052 18682327 PMC2860626

[19] Kuzuyama T, Noel JP, Richard SB. Structural basis for the promiscuous biosynthetic prenylation of aromatic natural products. Nature. 2005;435(7044):983–7. 10.1038/nature03668 15959519 PMC2874460

[20] Leipoldt F, Zeyhle P, Kulik A, Kalinowski J, Heide L, Kaysser L. Diversity of ABBA prenyltransferases in marine *Streptomyces* sp. CNQ-509: Promiscuous enzymes for the biosynthesis of mixed terpenoid compounds. PLoS One. 2015;10(12):1–15. 10.1371/journal.pone.0143237 PMC4684245 26659564

[21] Pojer F, Wemakor E, Kammerer B, Chen H, Walsh CT, Li S-M, et al CloQ, a prenyltransferase involved in clorobiocin biosynthesis. Proc Natl Acad Sci USA. 2003;100(5):2316–21. 10.1073/pnas.0337708100 12618544 PMC151338

[22] Bayse CA, Merz KM. Mechanistic insights into Mg^2+^-independent prenylation by CloQ from classical molecular mechanics and hybrid quantum mechanics/molecular mechanics molecular dynamics simulations. Biochemistry. 2014;53(30):5034–41. 10.1021/bi500531p 25020142

[23] Ozaki T, Mishima S, Nishiyama M, Kuzuyama T. NovQ is a prenyltransferase capable of catalyzing the addition of a dimethylallyl group to both phenylpropanoids and flavonoids. J Antibiot. 2009;62(7):385–92. 10.1038/ja.2009.48 19557032

[24] Cuyckens F, Claeys M. Mass spectrometry in the structural analysis of flavonoids. J Mass Spectrom. 2004;39(1):1–15. 10.1002/jms.585 14760608

[25] Aisyah S, Vincken J-P, Andini S, Mardiah Z, Gruppen H. Compositional changes in (iso)flavonoids and estrogenic activity of three edible *Lupinus* species by germination and *Rhizopus*-elicitation. Phytochemistry. 2016;122:65–75. 10.1016/j.phytochem.2015.12.015 26749476

[26] Kosters HA, Wierenga PA, de Vries R, Gruppen H. Characteristics and effects of specific peptides on heat-induced aggregation of β-lactoglobulin. Biomacromolecules. 2011;12(6):2159–70. 10.1021/bm2002285 21517078

[27] Metzger U, Schall C, Zocher G, Unsöld I, Stec E, Li S-M, et al The structure of dimethylallyl tryptophan synthase reveals a common architecture of aromatic prenyltransferases in fungi and bacteria. Proc Natl Acad Sci USA. 2009;106(34):14309–14. 10.1073/pnas.0904897106 19706516 PMC2732893

[28] Robert X G P. Deciphering key features in protein structures with the new ENDscript server. Nucleic Acid Research. 2014;42(W1):W320–W1. 10.1093/nar/gku316 PMC4086106 24753421

[29] Simons R, Vincken J-P, Bakx EJ, Verbruggen MA, Gruppen H. A rapid screening method for prenylated flavonoids with ultra-high-performance liquid chromatography/electrospray ionisation mass spectrometry in licorice root extracts. Rapid Commun Mass Spectrom. 2009;23(19):3083–93. 10.1002/rcm.4215 19711301

[30] van de Schans MGM, Vincken J-P, Bovee TFH, David Cervantes A, Logtenberg MJ, Gruppen H. Structural changes of 6a-hydroxy-pterocarpans upon heating modulate their estrogenicity. J Agric Food Chem. 2014;62(43):10475–84. 10.1021/jf503127c 25296697

[31] Yang M, Wang W, Sun J, Zhao Y, Liu Y, Liang H, et al Characterization of phenolic compounds in the crude extract of *Hedysarum multijugum* by high-performance liquid chromatography with electrospray ionization tandem mass spectrometry. Rapid Commun Mass Spectrom. 2007;21(23):3833–41. 17979101 10.1002/rcm.3277

[32] Morandi D, Le Quere JL. Influence of nitrogen on accumulation of isosojagol (a newly detected coumestan in soybean) and associated isoflavonoids in roots and nodules of mycorrhizal and non-mycorrhizal soybean. New Phytol. 1991;117(1):75–9.

[33] O'Neill MJ. Aureol and phaseol, two new coumestans from *Phaseolus aureus* Roxb. Z Naturforsch C. 1983;38(9–10):698–700.

[34] Stella L, De Rosso M, Panighel A, Vedova AD, Flamini R, Traldi P. Collisionally induced fragmentation of [M–H]^−^ species of resveratrol and piceatannol investigated by deuterium labelling and accurate mass measurements. Rapid Commun Mass Spectrom. 2008;22(23):3867–72. 10.1002/rcm.3811 18980255

[35] Sobolev VS, Potter TL, Horn BW. Prenylated stilbenes from peanut root mucilage. Phytochem Anal. 2006;17(5):312–22. 17019932 10.1002/pca.920

[36] Yang Y, Miao YP, Wang B, Cui GL, Merz KM. Catalytic Mechanism of Aromatic Prenylation by NphB. Biochemistry. 2012;51(12):2606–18. 10.1021/bi201800m 22385275 PMC3314166

