# Supplementary material for: Structural basis for non-genuine phenolic acceptor substrate specificity of Streptomyces roseochromogenes prenyltransferase CloQ from the ABBA/PT-barrel superfamily
Source: PLoS One. 2017 Mar 29;12(3):e0174665. doi: 10.1371/journal.pone.0174665 (PMC5371340; doi:10.1371/journal.pone.0174665)
Supplement: S1 File — (PDF) [file pone.0174665.s001.pdf]

Structural basis for non-genuine phenolic acceptor substrate specificity of *Streptomyces roseochromogenes* prenyltransferase CloQ from the ABBA/PT-barrel superfamily

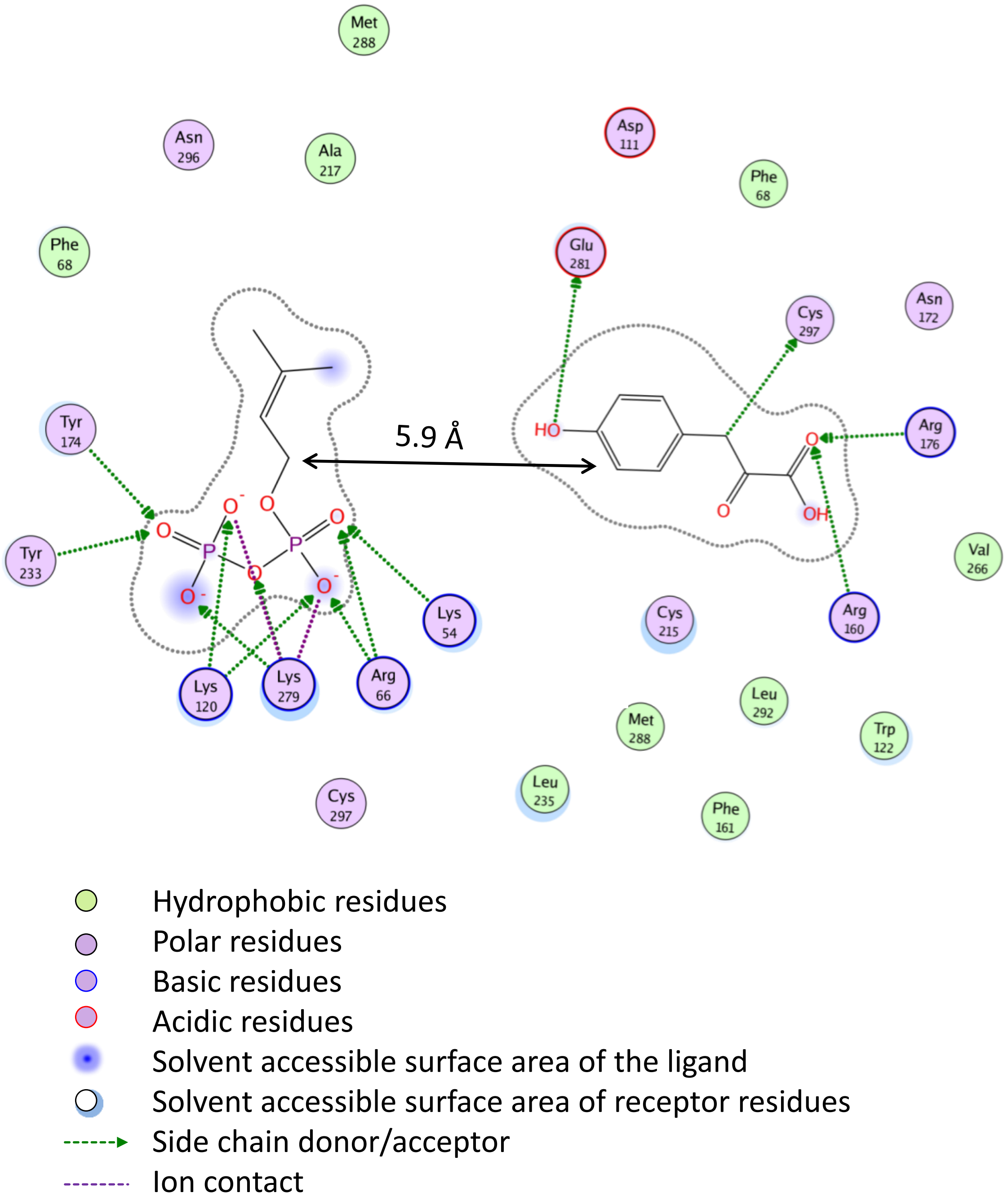

Figure A. Interaction 2D diagram between *Sr*CloQ and the genuine ligands 4-HPP (acceptor substrate) and DMAPP (donor substrate) as modelled in this study, using the PDB 2XLQ.

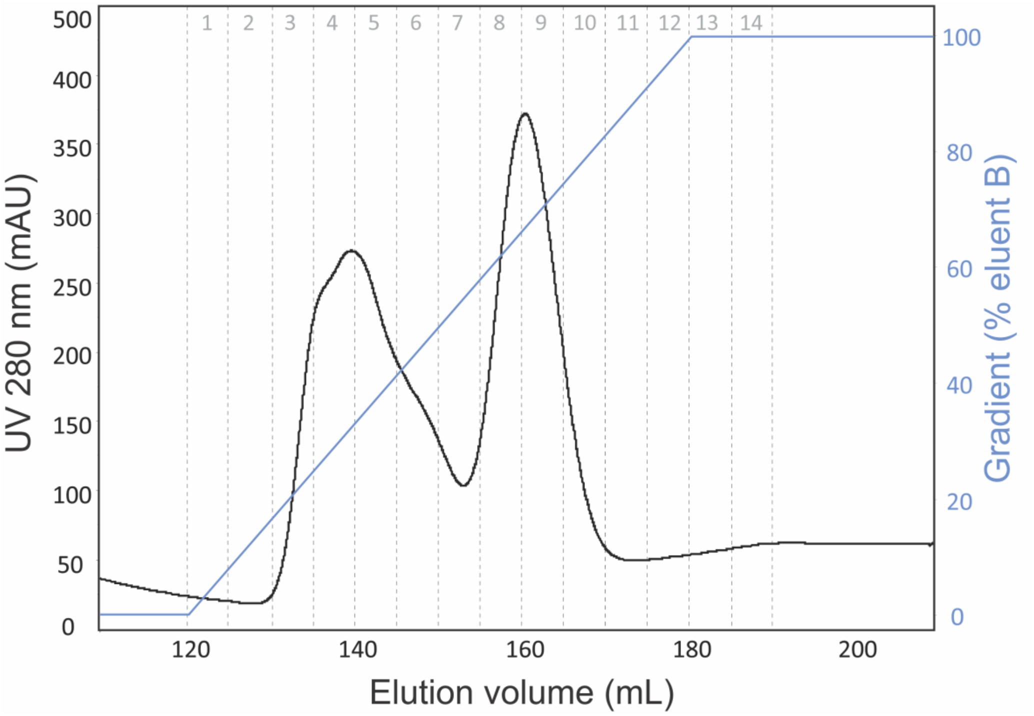

Figure B. Pattern of elution of the lysate for the purification of *Sr*CloQ by Ni^{2+} affinity chromatography. Eluent B, imidazole 250 mM in Tris-HCL buffer (pH 8.0). Peak at elution volume of 160 mL represents the His-tagged *Sr*CloQ protein. Grey numbers represent the fractions collected during the run. Fractions 8 and 9 were pooled.

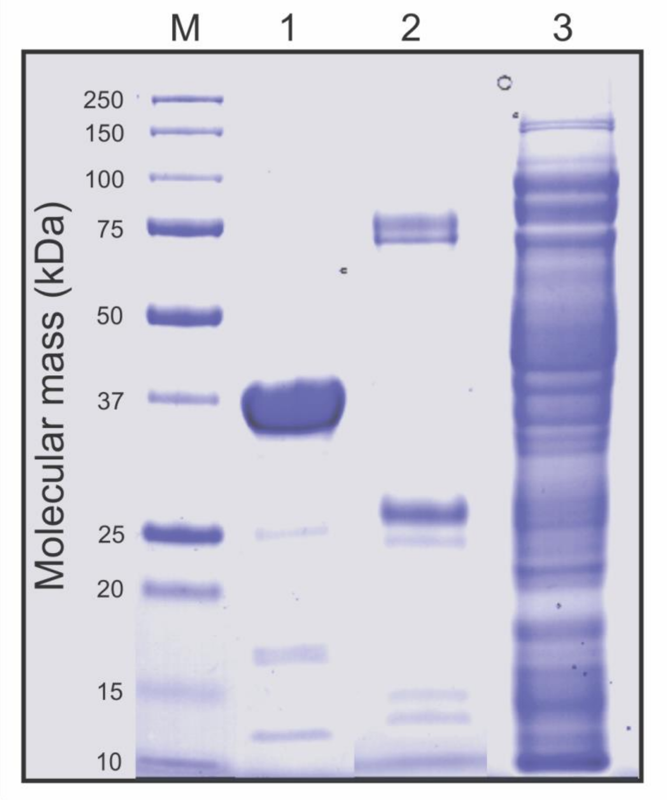

Figure C. SDS-PAGE of *Sr*CloQ enzyme. Lanes: M, molecular marker; 1, purified enzyme (35 kDa) after affinity chromatography and concentration (fractions 8-9, **Figure S1**); 2, non-*Sr*CloQ bound protein (fractions 4-6); 3, unbound protein (i.e. protein eluting before start of gradient).

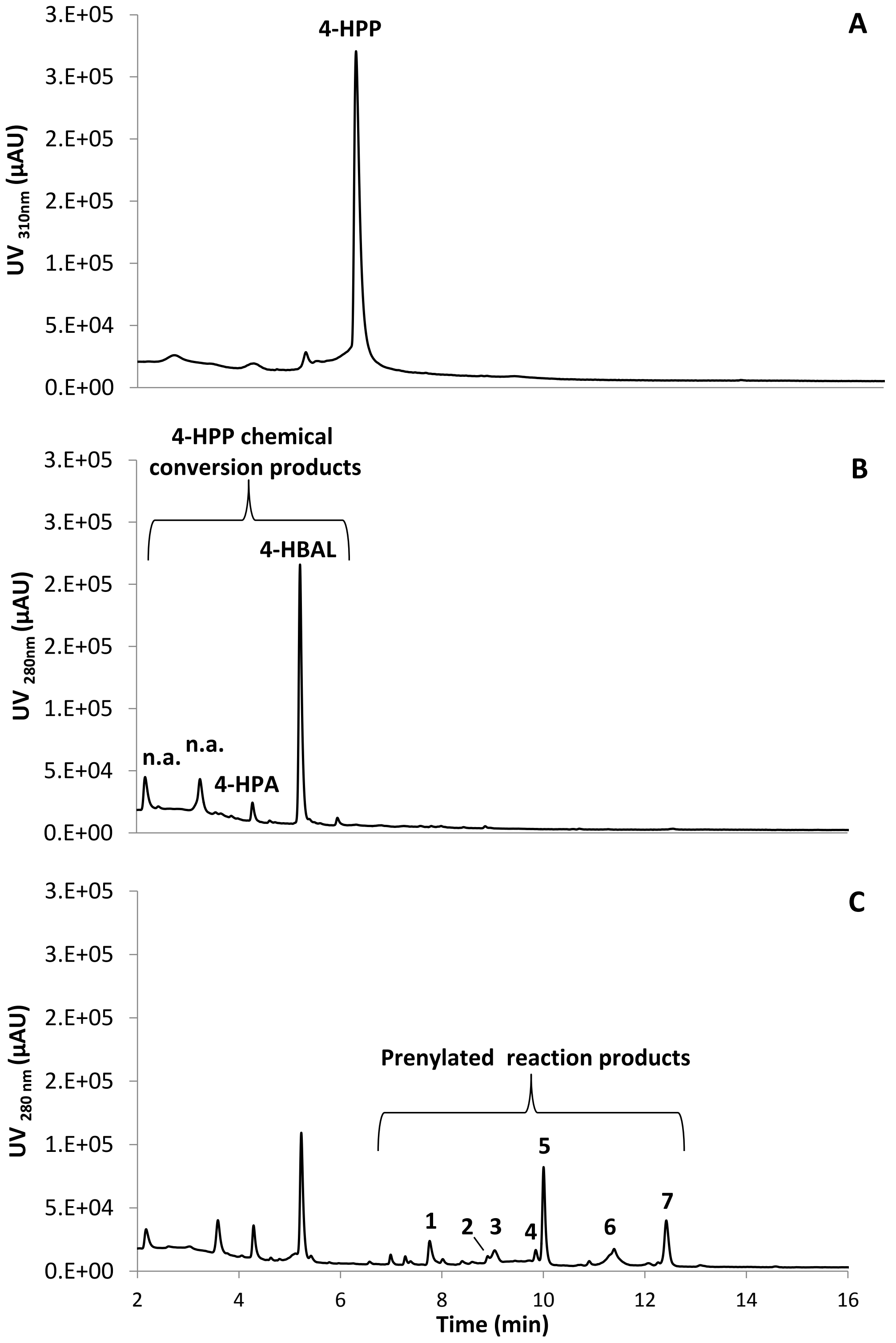

Figure D. UV chromatograms of 4-HPP standard solution (0.5 mM) (**A**), reaction products formed after incubation of 4-HPP and DMAPP without (**B**) and with *Sr*CloQ (**C**). Peak numbers refer to those from **Table 1** in the manuscript. 4-Hydroxybenzaldehyde (4-HBAL) and 4-hydroxyphenylacetic acid (4-HPA) were annotated using reference compounds.[1] Not annotated (n.a.).

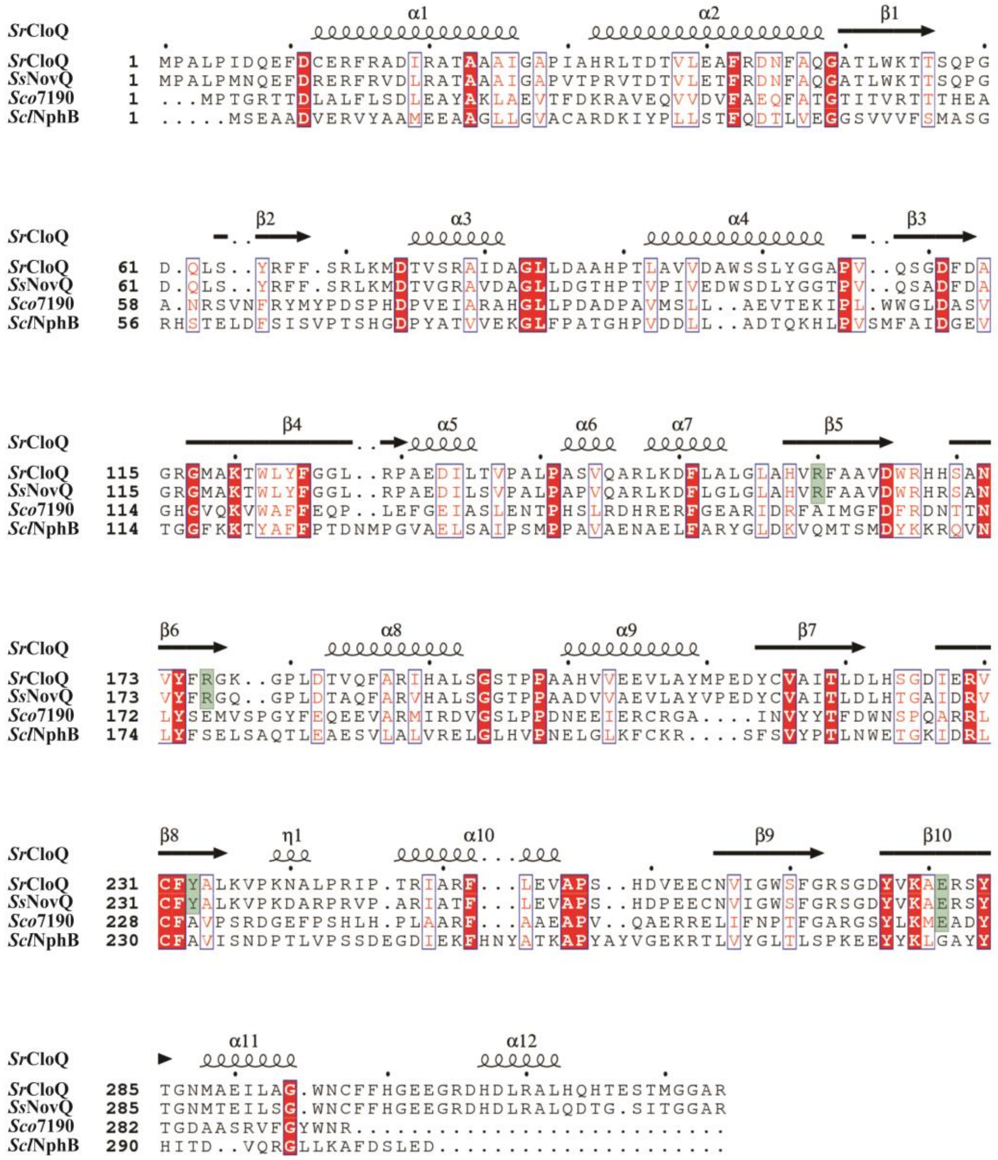

Figure E. Amino acid sequence alignment of PTs from the CloQ/NphB family. Strictly conserved residues are highlighted in red shaded boxes and highly similar residues are coloured in red and framed in blue. Important residues for (non-genuine) acceptor substrate binding in *Sr*CloQ, as discussed in this manuscript, are highlighted in green boxes. Secondary structure elements for *Sr*CloQ are shown above the alignment (α, α-helices; β, β-strands). Accession numbers: *Sr*CloQ, AAN65239; *Ss*NovQ, AAF67510; *Sci*7190, BAE00107; *Scl*NphB, BAE00106.

Most residues involved in binding of the donor substrate in the upper cavity of *Sr*CloQ (i.e. Asp111, Lys120, Asn172, Tyr174, Arg229, Tyr277, Lys279) [2] are identical in these members of the CloQ/NphB PT family. Residues providing the hydrophobic environment of the lower cavity of the PT barrel (e.g. Phe68, Trp122, Phe161, Val266 and Trp295 in *Sr*CloQ) were semi-conserved.

Table A. Molar extinction coefficients used for the quantification of prenylated products from the phenolic acceptor substrates.

Acceptor substrate	Wavelength (nm)	Molar extinction ($M^{-1}cm^{-1}$)	Reference
**Coumestrol**	345	24800	[3]
**Daidzein**	250	26915	[4]
**Equol**	281	6761	[5]
**Genistein**	263	31623	[4]
**Glycitein**	257	25388	[6]
**Isoflavone**	245	25704	[7]
**Luteolin**	256	15849	[4]
**Resveratrol**	308	29959	[8]
**3'-Hydroxy-daidzein**	250	15849	[9]
**4-Hydroxyphenylpyruvate**	276	2800	[10]
**7,4'-Dihydroxyflavone**	328	33000	[9]
**4'-Hydroxy-7-methoxy-flavone**	assumed to be similar as 7,4'-dihydroxyflavone[a]		
**4'-Hydroxy-6-methoxy-flavone**	assumed to be similar as 7,4'-dihydroxyflavone		

[a] Methoxylation has been shown not to significantly change the molar extinction coefficient of flavones.[11]

Structural basis for non-genuine phenolic acceptor substrate specificity of *Streptomyces roseochromogenes* prenyltransferase CloQ from the ABBA/PT-barrel superfamily

References

1. Doy CH. Alkaline conversion of 4-hydroxyphenylpyruvic acid to 4-hydroxybenzaldehyde. Nature. 1960;186(4724):529-31.
2. Metzger U, Keller S, Stevenson CEM, Heide L, Lawson DM. Structure and mechanism of the magnesium-independent aromatic prenyltransferase CloQ from the clorobiocin biosynthetic pathway. J Mol Biol. 2010;404(4):611-26.
3. Lee YJ, Notides AC, Tsay YG, Kende AS. Coumestrol, NBD-norhexestrol, and dansyl-norhexestrol, fluorescent probes of estrogen-binding proteins. Biochemistry. 1977;16(13):2896-901.
4. Weast RC. CRC Handbook of Chemistry and Physics. 58th ed. Boca Raton, FL, USA: CRC Press; 1977.
5. Franke AA, Lai JF, Halm BM. Absorption, distribution, metabolism, and excretion of isoflavonoids after soy intake. Arch Biochem Biophys. 2014;559:24-8.
6. Song TT, Hendrich S, Murphy PA. Estrogenic activity of glycitein, a soy isoflavone. J Agric Food Chem. 1999;47(4):1607-10.
7. Warburton WK. The isoflavones. Chem Soc Rev. 1954;8(1):67-87.
8. Trela BC, Waterhouse AL. Resveratrol: isomeric molar absorptivities and stability. J Agric Food Chem. 1996;44(5):1253-7.
9. Buckingham J, Munasinghe VRN. Dictionary of Flavonoids. Boca Raton, FL, USA: CRC Press; 2015. 843 p.
10. Johnson-Winters K, Purpero VM, Kavana M, Nelson T, Moran GR. (4-Hydroxyphenyl)pyruvate dioxygenase from *Streptomyces avermitilis*: the basis for ordered substrate addition. Biochemistry. 2003;42(7):2072-80.
11. Hartwig UA, Maxwell CA, Joseph CM, Phillips DA. Chrysoeriol and luteolin released from alfalfa seeds induce nod genes in *Rhizobium meliloti*. Plant Physiol. 1990;92(1):116-22.
